# Supplementary material for: Intestinal effect of faba bean fractions in WD-fed mice treated with low dose of DSS
Source: PLoS One. 2022 Aug 8;17(8):e0272288. doi: 10.1371/journal.pone.0272288 (PMC9359607; doi:10.1371/journal.pone.0272288)
Supplement: S1 Fig — Representative images of colon tissue from mice exposed to 1% DSS. Colon sections (7μm) were stained with hematoxylin and eosin. BF, both fractions; DSS, dextran sodium sulfate; FF, fiber fraction; PF, protein fraction; WD, western diet. (PDF) [file pone.0272288.s001.pdf]

S1 Fig.
Histology images

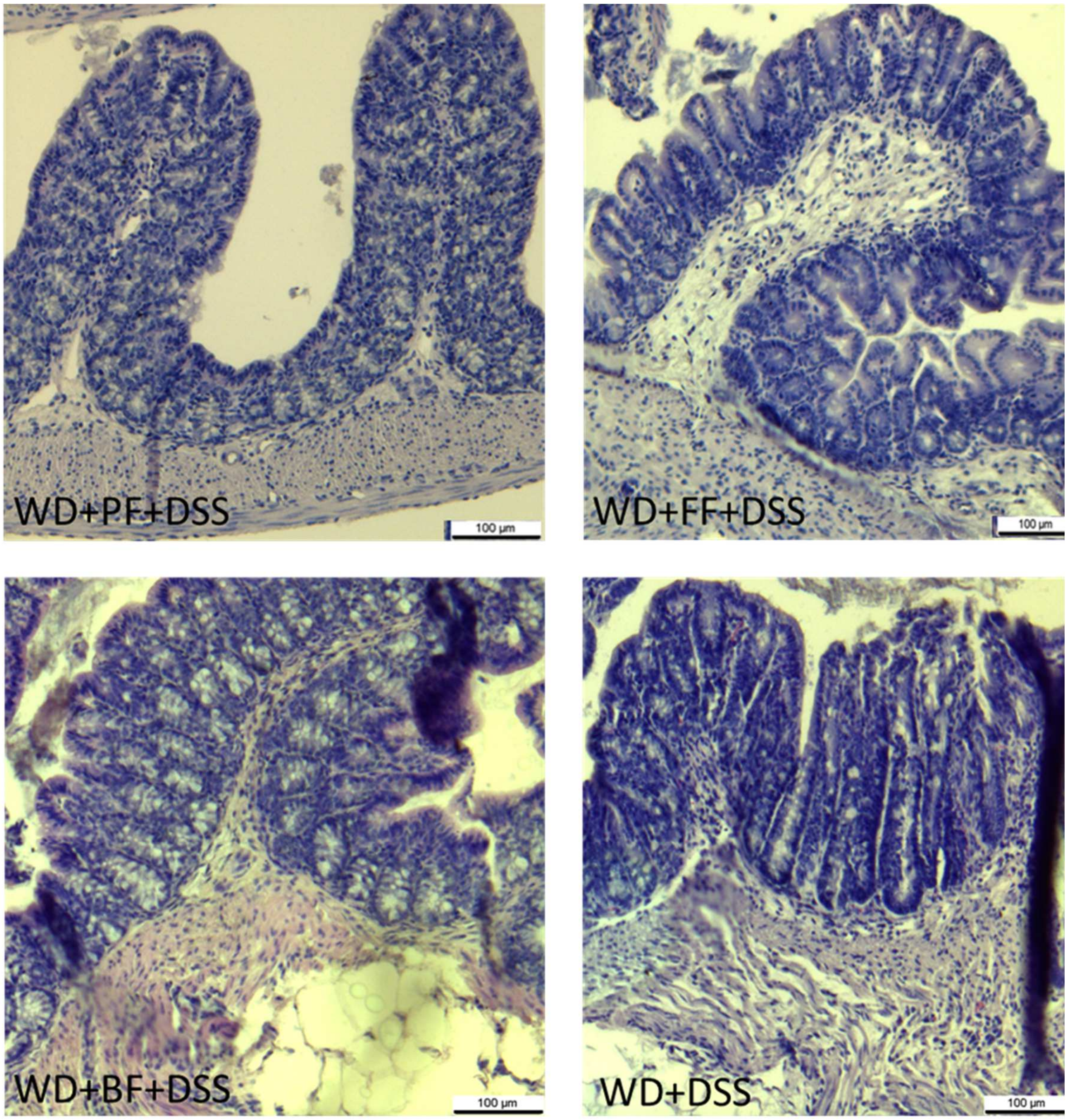

Supplementary figure 1. Representative images of colon tissue from mice exposed to 1% DSS. Colon sections (7µm) were stained with hematoxylin and eosin. BF, both fractions; DSS, dextran sodium sulfate; FF, fiber fraction; PF, protein fraction; WD, western diet.
