# Supplementary material for: Intestinal effect of faba bean fractions in WD-fed mice treated with low dose of DSS
Source: PLoS One. 2022 Aug 8;17(8):e0272288. doi: 10.1371/journal.pone.0272288 (PMC9359607; doi:10.1371/journal.pone.0272288)
Supplement: S1 Table — a. Composition of food pellets. b. Content of cooked faba bean fractions. (PDF) [file pone.0272288.s002.pdf]

S1 Table

a. Composition of food pellets

**Diets**	**WD**		**WD+PF**		**WD+FF**		**WD+BF**	
**Ingredients**	**g**	**kcal**	**g**	**kcal**	**g**	**kcal**	**g**	**kcal**
Casein	195	780	128.52	514	186.04	744.16	126.88	507.52
DL-methionine	3	12	3	12	3	12	3	12
Corn starch	50	200	38.91	155.64	45.46	181.84	36.07	144.28
Maltodextrin 10	100	400	100	400	100	400	100	400
Sucrose	341	1364	341	1364	341	1364	341	1364
Milk fat, cow, anhydrous	200	1800	196.95	1772	200	1800	197.22	1774
Corn oil	10	90	10	90	10	90	10	90
Cellulose, BW200	70	0	63.91	0	0	0	70	5.6
Ethoxyquin	0.04	0	0.04	0	0.04	0	0.04	0
Mineral mix S10001	35	0	35	0	35	0	35	0
Calcium carbonate	4	0	4	0	4	0	4	0
Vitamin mix V10001	10	40	10	40	10	40	10	40
Choline bitartrate	2	0	2	0	2	0	2	0
Cholesterol	1.5	0	1.5	0	1.5	0	1.5	0
Protein fraction	0	0	89.97	358.65	0	0	82	518.13
Fiber fraction	0	0	0	0	122.5	238.24	103	
Total	1021.5	4592.4	1024.8	4592.4	1060.5	4592.4	1057.3	4592.4
Protein, %	17.1	15.2	17.1	15.2	17.1	15.2	17.1	15.2
Carbohydrate, %	49	43.6	49	43.6	49	43.6	49	43.6
Fat, %	20.1	41.2	20.1	41.2	20.1	41.2	20.1	41.2
kcal/g	4.5		4.5		4.3		4.3	

BF, both fractions; FF, fiber fraction; PF, protein fraction; WD, Western diet (D12079B, Research Diets).

b. Content of cooked faba bean fractions

Sample	**Starch+Glucose+Fructose+Sucrose** Total available CHO (g/100g)	**NSP+NDO CHO** based Total Dietary Fiber (g/100g)	NDF (g/100g)	**Protein** (g/100g)	**Crude fat** (g/100g)
**Fiber fraction** (from hull)	3,70	57,14	62,68	6,44	0,27
**Protein fraction**	12,34	6,76	10,09	65,02	3,39

NSP: Non-starch polysaccharides, NDO: Non-digestible oligosaccharides, NDF: Neutral detergent fiber.
