# Supplementary material for: Intestinal effect of faba bean fractions in WD-fed mice treated with low dose of DSS
Source: PLoS One. 2022 Aug 8;17(8):e0272288. doi: 10.1371/journal.pone.0272288 (PMC9359607; doi:10.1371/journal.pone.0272288)
Supplement: S2 Table — (PDF) [file pone.0272288.s003.pdf]

S2 Table

Criteria for DAI and scoring way for assessing during exposure to 1% DSS.

	Day 0	Day 2		Day 4		Day 6 (termination)	
	Initial weight	Weight	% change from initial	Weight	% change from initial	Weight	% change from initial
Mouse 1							
Mouse 2							
Mouse 3							
Mouse 4							

	Activity		Hunched posture		Stool quality	
	Day 2	Day 4	Day 2	Day 4	Day 2	Day 4
Mouse 1						
Mouse 2						
Mouse 3						
Mouse 4						

	**For weight**	**For activity, hunched posture, stool quality**
**Scores given**	0=<5% weight loss	0: normal symptoms
	1= 5-15% weight loss	1: mild symptoms
	5= 15-20% weight loss	3: severe symptoms
	10=> 20% weight loss	
