# Supplementary material for: Intestinal effect of faba bean fractions in WD-fed mice treated with low dose of DSS
Source: PLoS One. 2022 Aug 8;17(8):e0272288. doi: 10.1371/journal.pone.0272288 (PMC9359607; doi:10.1371/journal.pone.0272288)
Supplement: S3 Table — (PDF) [file pone.0272288.s004.pdf]

S3 Table

Reaction mixture for cDNA synthesis using the iScript cDNA Synthesis kit (Bio Rad).

Component	Per reaction
5x iScript reaction mix	4 µL
iScript reverse transcriptase	1 µL
Nuclease-free water	11 µL
RNA template (200ng/µL)	4 µL
