# Supplementary material for: Intestinal effect of faba bean fractions in WD-fed mice treated with low dose of DSS
Source: PLoS One. 2022 Aug 8;17(8):e0272288. doi: 10.1371/journal.pone.0272288 (PMC9359607; doi:10.1371/journal.pone.0272288)
Supplement: S4 Table — (PDF) [file pone.0272288.s005.pdf]

S4 Table

Temperature program used for cDNA synthesis.

Operation	Temperature (°C)	Duration
Primer annealing	25	5 min
cDNA synthesis	42	30 min
cDNA synthesis termination	85	5 min
-	4	∞
