# Supplementary material for: Intestinal effect of faba bean fractions in WD-fed mice treated with low dose of DSS
Source: PLoS One. 2022 Aug 8;17(8):e0272288. doi: 10.1371/journal.pone.0272288 (PMC9359607; doi:10.1371/journal.pone.0272288)
Supplement: S5 Table — (PDF) [file pone.0272288.s006.pdf]

S5 Table

Primer sequences for RT-qPCR and melting temperatures.

Gene	Forward Primer 5'-3'	Reverse Primer 5'-3'	Tm °C
Gapdh	CTTCAACAGCAACTCCCACTCTT	GCCGTATTCATTGTCATACCAGG	60
Il-1b	GCAGCTGGAGAGTGTGGAT	AAACTCCACTTTGCTCTTGACTT	61
Il-6	CGTGGAAATGAGAAAAGAGTTGT	GCATCATCGTTGTTCATACA	61
Nos2	GACATTACGACCCCTCCCAC	ACTCTGAGGGCTGACACAAG	62
Nox2	GGGAACTGGGCTGTGAATGA	CAGTGCTGACCCAAGGAGTT	61
Ptgs2	AATATCAGGTCATTGGTGGAGA	TCTACCTGAGTGTCTTTGACTG	61
Tnf-a	CTGTCTACTGAACTTCGGGGTGAT	GGTCTGGGCCATAGAACTGATG	61

Abbreviations: *Gapdh***,** glyceraldehyde 3-phosphate dehydrogenase; *Il-1b*, interleukin-1 beta; *Il-6*, interleukin 6; *Nos2*, nitric oxide synthase 2; *Nox2*, NADPH oxidase 2; *Ptgs2*, prostaglandin endoperoxide synthase 2; *Tnf*-a, tumor necrosis factor alpha.
