# Supplementary material for: Intestinal effect of faba bean fractions in WD-fed mice treated with low dose of DSS
Source: PLoS One. 2022 Aug 8;17(8):e0272288. doi: 10.1371/journal.pone.0272288 (PMC9359607; doi:10.1371/journal.pone.0272288)
Supplement: S6 Table — (PDF) [file pone.0272288.s007.pdf]

S6 Table

Reaction mixture for amplicon PCR during library preparation for gene sequencing of 16S rRNA.

Component	Per reaction
5x HOT FIREPol® Blend Master Mix Ready to Load (Solis BioDyne)	5 µL
Forward primer, PRK341F (1 µM)*	0.5 µL
Reverse primer, PRK806R (1 µM)*	0.5 µL
Nuclease-free water	18 µL
Template DNA (0.003-2 ng/µL**)	1 µL

* Forward 5’- CCTACGGGRBGCASCAG-3’, reverse 5’- GGACTACYVGGGTATCTAAT-3’

** Measured by Qubit.
