# Supplementary material for: Intestinal effect of faba bean fractions in WD-fed mice treated with low dose of DSS
Source: PLoS One. 2022 Aug 8;17(8):e0272288. doi: 10.1371/journal.pone.0272288 (PMC9359607; doi:10.1371/journal.pone.0272288)
Supplement: S7 Table — (PDF) [file pone.0272288.s008.pdf]

S7 Table

Temperature cycles for amplicon PCR during library preparation for gene sequencing of 16S rRNA.

Operation	Temperature (°C)	Duration	Cycles
Initial activation	95	15 min	1
Denaturation	95	30 sec	
Annealing	55	30 sec	25
Elongation	72	45 sec	
Final elongation	72	7 min	1
-	4	∞	-
