# Supplementary material for: Intestinal effect of faba bean fractions in WD-fed mice treated with low dose of DSS
Source: PLoS One. 2022 Aug 8;17(8):e0272288. doi: 10.1371/journal.pone.0272288 (PMC9359607; doi:10.1371/journal.pone.0272288)
Supplement: S8 Table — (PDF) [file pone.0272288.s009.pdf]

S8 Table

Reaction mixture for index PCR during library preparation for gene sequencing of 16S rRNA.

Component	Per reaction
5x FIREPol® Master Mix Ready to Load (Solis BioDyne)	5 µL
Forward primer (1 µM)*	5 µL
Reverse primer (1 µM)*	5 µL
Nuclease-free water	8 µL
Template DNA	2 µL

* See Supplementary table 10
