# Supplementary material for: Intestinal effect of faba bean fractions in WD-fed mice treated with low dose of DSS
Source: PLoS One. 2022 Aug 8;17(8):e0272288. doi: 10.1371/journal.pone.0272288 (PMC9359607; doi:10.1371/journal.pone.0272288)
Supplement: S10 Table — (PDF) [file pone.0272288.s011.pdf]

S10 Table

Primers modified with Illumina adapters used for index PCR during library preparation for gene sequencing of 16S rRNA. Unique combination of forward and reverse primer was used for each sample.

Primer name	Sequence, 5' -> 3'	Target region	Direction
**F9**	aatgatacggcgaccaccgagatctacactctttccctacacgacgctcttccgatctgtttcgCCTACGGGRBGCASCAG	16S rRNA (V3-V4)	Forward
**F10**	aatgatacggcgaccaccgagatctacactctttccctacacgacgctcttccgatctcgtacgCCTACGGGRBGCASCAG	16S rRNA (V3-V4)	Forward
**F11**	aatgatacggcgaccaccgagatctacactctttccctacacgacgctcttccgatctgagtggCCTACGGGRBGCASCAG	16S rRNA (V3-V4)	Forward
**F12**	aatgatacggcgaccaccgagatctacactctttccctacacgacgctcttccgatctggtagcCCTACGGGRBGCASCAG	16S rRNA (V3-V4)	Forward
**F13**	aatgatacggcgaccaccgagatctacactctttccctacacgacgctcttccgatctactgatCCTACGGGRBGCASCAG	16S rRNA (V3-V4)	Forward
**F14**	aatgatacggcgaccaccgagatctacactctttccctacacgacgctcttccgatctatgagcCCTACGGGRBGCASCAG	16S rRNA (V3-V4)	Forward
**F15**	aatgatacggcgaccaccgagatctacactctttccctacacgacgctcttccgatctattcctCCTACGGGRBGCASCAG	16S rRNA (V3-V4)	Forward
**F16**	aatgatacggcgaccaccgagatctacactctttccctacacgacgctcttccgatctcaaaagCCTACGGGRBGCASCAG	16S rRNA (V3-V4)	Forward
**R25**	caagcagaagacggcatacgagatATCAGTgtgactggagttcagacgtgtgctcttccgatctGGACTACYVGGGTATCTAAT	16S rRNA (V3-V4)	Reverse
**R26**	caagcagaagacggcatacgagatGCTCATgtgactggagttcagacgtgtgctcttccgatctGGACTACYVGGGTATCTAAT	16S rRNA (V3-V4)	Reverse
**R27**	caagcagaagacggcatacgagatAGGAATgtgactggagttcagacgtgtgctcttccgatctGGACTACYVGGGTATCTAAT	16S rRNA (V3-V4)	Reverse
**R28**	caagcagaagacggcatacgagatCTTTTGgtgactggagttcagacgtgtgctcttccgatctGGACTACYVGGGTATCTAAT	16S rRNA (V3-V4)	Reverse
**R29**	caagcagaagacggcatacgagatTAGTTGgtgactggagttcagacgtgtgctcttccgatctGGACTACYVGGGTATCTAAT	16S rRNA (V3-V4)	Reverse
**R30**	caagcagaagacggcatacgagatCCGGTGgtgactggagttcagacgtgtgctcttccgatctGGACTACYVGGGTATCTAAT	16S rRNA (V3-V4)	Reverse
**R31**	caagcagaagacggcatacgagatATCGTGgtgactggagttcagacgtgtgctcttccgatctGGACTACYVGGGTATCTAAT	16S rRNA (V3-V4)	Reverse
**R32**	caagcagaagacggcatacgagatTGAGTGgtgactggagttcagacgtgtgctcttccgatctGGACTACYVGGGTATCTAAT	16S rRNA (V3-V4)	Reverse
**R33**	caagcagaagacggcatacgagatCGCCTGgtgactggagttcagacgtgtgctcttccgatctGGACTACYVGGGTATCTAAT	16S rRNA (V3-V4)	Reverse
**R34**	caagcagaagacggcatacgagatGCCATGgtgactggagttcagacgtgtgctcttccgatctGGACTACYVGGGTATCTAAT	16S rRNA (V3-V4)	Reverse
**R35**	caagcagaagacggcatacgagatAAAATGgtgactggagttcagacgtgtgctcttccgatctGGACTACYVGGGTATCTAAT	16S rRNA (V3-V4)	Reverse
