# Supplementary material for: Intestinal effect of faba bean fractions in WD-fed mice treated with low dose of DSS
Source: PLoS One. 2022 Aug 8;17(8):e0272288. doi: 10.1371/journal.pone.0272288 (PMC9359607; doi:10.1371/journal.pone.0272288)
Supplement: Attachment — Submitted filename: Response to Reviewers.docx [file pone.0272288.s012.docx]

**Papoutsis et al., “Intestinal effect of faba bean fractions in WD-fed mice treated with low dose of DSS”**

**Response to editor and reviewer**

**Editor’s comments:**

1. Please ensure that your manuscript meets PLOS ONE's style requirements, including those for file naming. The PLOS ONE style templates can be found at <https://journals.plos.org/plosone/s/file?id=wjVg/PLOSOne_formatting_sample_main_body.pdf> and 
<https://journals.plos.org/plosone/s/file?id=ba62/PLOSOne_formatting_sample_title_authors_affiliations.pdf>

**Reply:** PLOS ONE style templates were taken into consideration and applied in order to meet journal’s requirements.

2. Please further describe the source of the Faba beans used in this study.

**Reply:** We agree that some information was missing. We have therefore included this information in the Method section: “The faba bean strain, Vertigo, was grown and harvested in Denmark and shipped to Norway for further processing”. This is now incorporated in the manuscript (Lines 107-108).

3. PLOS ONE now requires that authors provide the original uncropped and unadjusted images underlying all blot or gel results reported in a submission’s figures or Supporting Information files. This policy and the journal’s other requirements for blot/gel reporting and figure preparation are described in detail at <https://journals.plos.org/plosone/s/figures#loc-blot-and-gel-reporting-requirements> and <https://journals.plos.org/plosone/s/figures#loc-preparing-figures-from-image-files>. When you submit your revised manuscript, please ensure that your figures adhere fully to these guidelines and provide the original underlying images for all blot or gel data reported in your submission. See the following link for instructions on providing the original image data: <https://journals.plos.org/plosone/s/figures#loc-original-images-for-blots-and-gels>. 
  
In your cover letter, please note whether your blot/gel image data are in Supporting Information or posted at a public data repository, provide the repository URL if relevant, and provide specific details as to which raw blot/gel images, if any, are not available. Email us at [plosone@plos.org](mailto:plosone@plos.org) if you have any questions.

**Reply:** No blot/gel images are included in our manuscript.

4. We note that you have included the phrase “data not shown” in your manuscript. Unfortunately, this does not meet our data sharing requirements. PLOS does not permit references to inaccessible data. We require that authors provide all relevant data within the paper, Supporting Information files, or in an acceptable, public repository. Please add a citation to support this phrase or upload the data that corresponds with these findings to a stable repository (such as Figshare or Dryad) and provide and URLs, DOIs, or accession numbers that may be used to access these data. Or, if the data are not a core part of the research being presented in your study, we ask that you remove the phrase that refers to these data.

**Reply:** The phrase “data not shown” in the manuscript has been removed and the relevant text in the manuscript has been revised.

**Reviewers' comments and responses to questions:**

**1.** Is the manuscript technically sound, and do the data support the conclusions?

The manuscript must describe a technically sound piece of scientific research with data that supports the conclusions. Experiments must have been conducted rigorously, with appropriate controls, replication, and sample sizes. The conclusions must be drawn appropriately based on the data presented.

Reviewer #1: Partly

**Reply:** Indeed, we are aware of certain shortcomings of the study. In the discussion, we have indicated that we should have included data on microbiota composition without DSS. However, this was not the scope of this study. Nevertheless, in hindsight, we wish we had done that.

**2.** Has the statistical analysis been performed appropriately and rigorously?

Reviewer #1: Yes

**Reply:** Thank you for the kind comment.

**3.** Have the authors made all data underlying the findings in their manuscript fully available?

The [PLOS Data policy](http://www.plosone.org/static/policies.action#sharing) requires authors to make all data underlying the findings described in their manuscript fully available without restriction, with rare exception (please refer to the Data Availability Statement in the manuscript PDF file). The data should be provided as part of the manuscript or its supporting information, or deposited to a public repository. For example, in addition to summary statistics, the data points behind means, medians and variance measures should be available. If there are restrictions on publicly sharing data—e.g. participant privacy or use of data from a third party—those must be specified.

Reviewer #1: Yes

**Reply:** Thank you.

**4.** Is the manuscript presented in an intelligible fashion and written in standard English?

PLOS ONE does not copyedit accepted manuscripts, so the language in submitted articles must be clear, correct, and unambiguous. Any typographical or grammatical errors should be corrected at revision, so please note any specific errors here.

Reviewer #1: Yes

**Reply:** Thanks a lot.

**5.** Review Comments to the Author

Please use the space provided to explain your answers to the questions above. You may also include additional comments for the author, including concerns about dual publication, research ethics, or publication ethics. (Please upload your review as an attachment if it exceeds 20,000 characters)

Reviewer #1: Ms. ID PONE-D-22-03545: "Intestinal effect of faba bean fractions in WD-fed mice treated with low dose of DSS"


General comments:
The aim of the present study was to determine the ability aba bean fractions to reduce low-grade inflammation induced by a Western diet (High Fat diet) and the addition of DSS during the last six days before the end of experiment, and to modulate the intestinal microbiota of male C57BL/6JRj mice after 8 weeks. Wester diet (WD) and Protein Fraction (PF) diet increased the weight gain as compared to WD and WD+ Fiber Fraction (FF) and no signification difference was observed for glucose regulation. Protein and fiber fraction from faba beans did not alleviate the effect of low-grade colonic inflammation induced by the 1% DSS treatment. Faba bean fractions seems able to modulate gut microbiota. Therefore, the authors concluded that the replacement of protein and or fiber in WD with those of faba bean fraction had marginal effects on colonic and inflammation parameters. The role of faba bean fractions as gut microbiota modulator was not clearly demonstrated although fiber fraction seems to modulate the relative abundance of Alloprevotella and Prevotellaceae.

This study is built on basic analysis and mice model that provides preliminary information on the effects of faba bean fractions on alleviation of low-grade inflammation induced by DSS and their impact on modulation of gut microbiota.

**Reply:** Thank you for the general comment. Initially we thought that faba bean fractions would affect microbiota to a greater extent than what we observed. We believe, though, that the scrutinization of the bacterial composition was thorough and reflects what we found. A challenge is that the effects of the two fractions was not easily separated since they were not pure, i.e. the protein fraction also contained other nutrients than proteins including some fibers and phytochemicals that could affect microbiota.

**Specific comments from reviewer:
Abstract**
**Line 46:** but modulated intestinal microbiota. Comments: The results are not as convincing; it seems that the fiber fraction can modulate members of the Prevotella family. However, this same fraction reduces the relative abundance of bifidobacteria. It would be more prudent to point out that the faba fractions also had marginal effects on intestinal microbiota.

**Reply:** Thank you. We agree with the comment. The proposal made is accepted and the text has been revised (Line 34-35).

**Introduction**
**Lines 90-94:** Mice fed a western diet may develop low-grade inflammation. This low-grade inflammation can also be simulated by inducing it with a DSS intervention. Here, the authors chose to do both. A rationale for this model would be needed or supported by references (see lines 354-356, no reference).

**Reply:** It may seem redundant to include two types of “stressors”. However, the two “stressors” represent two different situations. A western diet/high fat diet represents in our opinion a realistic diet common to a large proportion of the population, which only manifests itself as a marginal inducer of inflammation. DSS in low doses, on the other hand represents another type of realistic trigger of intestinal inflammation, and the idea is that a low dose of DSS would simulate episodes of inflammatory stimuli represented by minor transient intestinal damages, IBD in remission, IBS or transient presence of opportunistic or pathogenic bacteria. The hypothesis was therefore that a western diet increased vulnerability towards DSS that could potentially be ameliorated/rescued by faba beans. Indeed, several studies in both humans and mice have shown that either a high fat diet or western diet creates an inflammatory environment associated with IBD or similar inflammatory condition. This has been reviewed by Christ et al. Immunity 2019. This reference has therefore been inserted in the manuscript (Line 346).

**Results**
**Line 330-331:** Please specify higher relative abundance levels instead of higher levels. In addition, these results should be discussed as higher relative abundance levels of Proteobacteria and mucin-degrading bacteria (Verrucomicrobia) are observed in experiments that sum up DSS-induced chronic inflammations, suggesting a dysbiosis. The fractions studied would therefore not have an effect to reduce this dysbiosis.

**Reply:** The phrase higher levels has been modified and replaced with higher relative abundance levels as suggested. Additionally, the sentence referred to above may be unclear. Firstly, all DSS treated WD-fed (including those supplemented with faba bean fractions) manifested higher relative abundance of Proteobacteria and Verrucomicrobia compared to the untreated WD-fed mice (no DSS). However, the relative abundance of those two phyla was highest in the DSS treated mice fed with the WD solely. Mice fed a WD supplemented with faba bean fractions revealed lower levels of Proteobacteria and Verrucomicrobia. Although most comparisons between WD+DSS mice and WD+ faba bean fractions+ DSS mice (except the WD+PF fed mice with *P*=0.04) showed no statistical significance but only a tendency, it cannot be stated that faba bean fractions had no impact on the microbiota profile. Therefore, the sentences have been revised (Lines 316, 318-322).

**Discussion**
Lines 402-407: The comment on the higher relative abundance of proteobacteria and mucin-degrading bacteria observed in DSS treated WD mice should be discussed.

**Reply:** We agree that we should discuss this more. The phylum Verrucomicrobia, which has only one representative in the human and mouse gut (Akkermansia muciniphila) is characterized by mucin degrading properties. The role of this genus is debated since some studies support that it contributes beneficially to intestinal homeostasis (Derrien et al., 2011), whereas other studies demonstrate involvement in exacerbating intestinal inflammation (Zhang *et al.,* 2021). The same conflicts for *Akkermansia* appear regarding its beneficial (Shin *et al.,* 2014) or harmful effect (Zhang et al, 2016) in mice fed high fat diets.

Administration of DSS led to a rise of Proteobacteria, which agrees with other studies (Chang et al., 2021). The phylum Proteobacteria is an indicator of an inflammatory phenotype (Shin et al., 2015). Higher abundance of Proteobacteria and reduced diversity of the gut microbiome have become increasingly common in cases of IBD (Berry and Reinisch, 2013).

A discussion in line with the above has been inserted into lines 403-420.

**6.** PLOS authors have the option to publish the peer review history of their article ([what does this mean?](https://journals.plos.org/plosone/s/editorial-and-peer-review-process#loc-peer-review-history)). If published, this will include your full peer review and any attached files.

If you choose “no”, your identity will remain anonymous but your review may still be made public.

**Do you want your identity to be public for this peer review?** For information about this choice, including consent withdrawal, please see our [Privacy Policy](https://www.plos.org/privacy-policy).

Reviewer #1: No
